# Prevalence and determinants of under-five mortality (U5M) in low-and lower -middle-income countries: Evidence from recent demographic and health surveys

**DOI:** 10.1371/journal.pone.0336616

**Published:** 2025-11-20

**Authors:** Md. Alamgir Sarder, Tabassum Mehedi, Benojir Ahammed, Md. Khairul Islam, Subarna Kundu, Murfia Muna, Maliha Mahajabin

**Affiliations:** 1 Statistics Discipline, Science, Engineering and Technology School, Khulna University, Khulna, Bangladesh; 2 School of Business and Centre for Health Research, University of Southern Queensland, Toowoomba, Australia; The University of Queensland, AUSTRALIA

## Abstract

**Objectives:**

Under-five mortality (U5M) remains a major global challenge, particularly in low-and lower-middle- income countries (LLMICs) where healthcare disparities are prevalent. This study evaluates the prevalence of U5M and examines the relative importance of its associated factors across 32 LLMICs.

**Methods:**

Data from the Demographic and Health Survey (2011–2024) were used for analysis. A total of 266,333 children under five years of age were included to assess the association of 20 factors at both individual and community levels with U5M. The chi-square test and multivariate logistic regression model were applied to determine the association of these factors with U5M.

**Results:**

Among the children aged 0−59 months, the prevalence of U5M was 40.5 per 1,000 (95% CI: 39.8–41.2). In the pooled sample, at the individual level, family member (>7) was the strongest factor associated with U5M (adjusted odds ratio (AOR):1.27; 95% CI:1.22–1.33, p < 0.001), followed by maternal age ≥ 35 years (AOR: 1.25; 95% CI:1.20–1.30, p < 0.001), being a female child (AOR:1.19; 95% CI:1.15–1.24, p < 0.001), and maternal unemployment (AOR:1.10; 95% CI:1.05–1.14, p < 0.001). At the community level, high maternal illiteracy (AOR:1.52; 95% CI:1.35–1.70, p < 0.001) was the most significant factor, followed by rural residence (AOR:1.26; 95% CI:1.15–1.38, p < 0.001), high paternal illiteracy (AOR:1.20; 95% CI:1.10–1.30, p < 0.001), and high maternal unemployment (AOR: 1.10; 95% CI:1.02–1.20, p < 0.001). Furthermore, large family members at individual levels and high maternal illiteracy at the community level consistently rank among the top two strongest factors across most countries, with a few exceptions.

**Conclusions:**

In LLMICs, U5M remains high, strongly associated with large family size and high maternal illiteracy. Governments and non-governmental organizations should promote maternal education and contraception use to facilitate birth spacing and family planning, while tailoring interventions to country-specific contexts.

## Introduction

Globally, children’s under-five mortality (U5M) remains unsolved public health problems, especially in low-and lower-middle-income countries (LLMICs) [1]. One of the main objectives of the Sustainable Development Goals (SDGs) is to reduce mortality, and SDG 3.2 specifically addresses the mortality of children under five [2]. Significant drops in current rates in LLMICs will be necessary to achieve these goals, where the great majority of child deaths currently take place [3]. In 2021 alone, approximately 5 million children under the age of five died worldwide, equating to around 13,800 deaths per day an unacceptably high toll of largely preventable child mortality. The probability of a child dying before completing five years of age is still the highest in LLMICs [4]. In lower-middle-income and upper-middle-income Asia-Pacific countries, the average under-five mortality rate of children was 33.7 and 11.7 deaths per 1,000 live births, respectively [5]. Over the previous seven decades, U5M rates in Africa have steadily declined. Between 1955 and 2020, child mortality rates were estimated to have decreased from 311 to 71 deaths per thousand live births [6]. However, the rate of decline varies between countries. Sub-Saharan Africa and Southeast Asia bear the highest burden of U5M [7–9].

Over the past three decades, there has been a significant global improvement in child survival rates. In 2021, 1 in 26 children died before reaching the age of five, compared to 1 in 11 in 1990, giving millions of children a much higher chance of survival than they had in the past [10]. In addition, the 2000s saw a faster pace of improvement in reducing child mortality rates compared to the 1990s. The global U5M rate decreased at an annual rate of 2.7% from 2010 to 2021, and 4.0% from 2000 to 2009, up from 1.8% in the 1990s [11]. While the global rate of U5M has significantly decreased, several LLMICs have not experienced this same fall. Niger led the world in the number of deaths of children under five in 2021, with an average of about 115 deaths per 1,000 live births [12]. According to 2023 report, the U5M rate in eight countries across Asia and the Pacific remained higher in 2021 than the global average of 38.1 deaths per 1,000 live births [13].

Although numerous studies on U5M have been conducted, most have focused on identifying specific factors relevant to individual countries or subregions. However, LLMICs share similar social, demographic, and economic context, suggesting that the determinants of U5M are likely comparable across these regions.

Several individual and community-level factors contribute to U5M rates in LLMICs. In rural areas, U5M is closely linked to addressing early marriage and teenage pregnancies, as young mothers face higher risks of anemia, undernutrition, and pregnancy complications. These challenges often lead to adverse child outcomes such as low birth weight, prematurity, feeding difficulties, cognitive impairments, and asthma [14–20]. Poverty is a well-documented determinant of U5M, with rates generally higher among poorer households, although some studies report greater U5M among wealthier groups [21–25]. Limited media access and inadequate water and sanitation further exacerbate U5M rates in these regions [26,27]. Existing literature indicates that U5M is independently influenced by factors such as family size [26–28]. Mixed results have been reported for maternal age [26,29], sex of children [27,30], and women’s working status [31]. Additional determinants maternal education [26,27,30–32], father’s education [26,27,33–35], cesarean delivery [31,36–38], birth order [30–32,39,40], and maternal age at first birth [30,40]. However, few studies examined community-level factors such as rural residence [26,29–31], community poverty, parental education, and media access [26,41] in the context of U5M across LLMICs. To the best of our knowledge, previous studies have not investigated the relative ranking of individual- and community-level factors associated with U5M across LLMICs. The main aim of this study is to identify these factors, examine their associations with U5M and compare their relative importance to determine the most influential predictors across LLMICs.

## Methods and methodology

### Study design, setting, and participants

The study utilized data from the latest rounds of Demographic and Health Surveys (DHS) conducted across 32 low- and lower-middle-income countries (LLMICs) between 2011 and 2024. Data were obtained from the children’s file, including the children aged 0–59 months. The DHS provides nationally representative data based on interviews with women aged 15–49 years, using a two-stage stratified cluster sampling design. In the first stage, a predetermined number of primary sampling units (PSUs) were selected with probability proportional to size, followed by systematic random sampling of 30 households within each PSU [42–44]. Information was collected using structured questionnaires administered by trained interviewers, covering respondents’ sociodemographic characteristics, individual and community factors, and a range of health-related indicators. National agencies collaborate with international organizations, such as ICF, to conduct DHS surveys, ensuring a combination of local oversight and standardized global methodology. Detailed summaries of each DHS survey are available, and all datasets are publicly accessible upon request at: https://dhsprogram.com/data/dataset_admin/login_main.cfm. A total of 266,333 children met the inclusion criteria, and 2,311,115 were excluded due to missing responses (e.g., refusals or absences) or biologically improbable anthropometric measurements.

### Outcome variable

The dependent variable in this study is under-five mortality (U5M). This variable was coded as a binary outcome: 1 (Child died before age five) and 0 (Child survived beyond age five). Information on births and deaths was obtained from the DHS calendar, which records monthly data on births and deaths. For women with multiple children, each child was considered as a separate observation and assigned an individual outcome status. This outcome variable was utilized to investigate the determinants associated with U5M in LLMICs.

### Independent variables

This study included several independent variables, selected based on previous literature and data availability [26–32]. For the pooled analysis, twelve individual-level explanatory variables were considered: maternal age (15–24, 25–34, 35–49 years), wealth index (poor, middle, rich), media access (no, yes), maternal education (no formal education, primary, secondary and higher), fathers education (no formal education, primary, secondary and higher), maternal age at birth (<19 years, ≥ 19 years), working status (unemployed, employed), vaginal delivery (yes, no), sex of child (male, female), family member (≤7, > 7), birth order (≤3, > 3), and drinking water (unimproved, improved).

At the community level, eight variables namely community poverty, maternal illiteracy, fathers’ illiteracy, media inaccessibility, maternal unemployment, vaginal delivery, and unimproved water sources were generated by aggregating individual-level factors within each cluster except for residence. Cluster-level percentages were used to categorize communities into high- and low-incidence groups. Finally, for ranking potential determinants of U5M, all independent variables were dichotomous (yes/no), including maternal age (≥35 years), poor wealth index, media inaccessibility, maternal no formal/primary education, fathers no formal education/primary education, maternal age at birth (<19 years), maternal unemployment, vaginal delivery, female sex of the child, large household size (>7), higher birth order (>3), and unimproved drinking water source.

### Statistical analysis

Data from all included countries were pooled, and the effect of each factor on U5M was examined. To ensure nationally representative and valid in pooled estimates, sampling weights, clustering, and stratification variables provided by DHS were incorporated. The weighted prevalence of U5M was calculated as the number of deaths (numerator) before age five per 1,000 live births (denominator). Descriptive analyses, including frequency distributions, weighted prevalence, and sample size distributions, were used to summaries the basic characteristics of respondents across all LLMICs. The Chi-square test was conducted to assess the bivariate associations between covariates and U5M. Two sets of logistic regression models were estimated: one using pooled data, and another using country-specific data. Factors were ranked based on the magnitude of their coefficient (odds ratios [ORs]) obtained from these models. The best-off group served as the reference category for all variables to maintain consistency in the interpretation of ORs. For country-specific analyses, the relative ranking of individual- and community-levels factors was displayed separately. Multicollinearity was assessed using the variance inflation factor (VIF), and all VIF values were below the commonly accepted threshold of 10, indicating no evidence of serious multicollinearity. Study reliability was supported by applying consistent modeling procedure and comparing consistent results across polled and country-specific analysis. Data analysis was conducted using Microsoft Excel, IBM SPSS Statistics (version 25), and Stata (version 17, StataCorp). All statistical tests were two-tailed, with statistical significance defined as *p* < 0.05.

### Ethical approval

This study was carried out using secondary data analysis from the MEASURE demography, health and survey (DHS). This survey confirmed globally ethical standards during surveys that received ethical clearance from both the ORC Macro Inc. Ethics Committee and the Ministry of Health Ethics Boards of the 32 LLMICs.

## Results

### Baseline characteristics

Table 1 presents the sample size distribution of children aged 0–59 months included in the analysis of U5M across LLMICs using DHS data from 2011 to 2024. A total of 266,333 children aged 0–59 months were included in the analysis. Sample sizes varied considerably by country, ranging from 1,002 in Lesotho to 33,019 in India. However, Liberia exhibited the highest U5M rate, with 78 deaths per 1,000 live births, while Peru reported the lowest rate, with 18 per 1,000 live births.

**Table 1:** Sample size and distribution of children aged 0–59 months included in the study of U5M across 32 LLMICs, DHS 2011–2024.

Country	Year of survey	Sample size	Number of U5M	U5M per 1000 live birth
Afghanistan	2015	31,832	1,585	46
Angola	2015−16	9,243	450	52
Bangladesh	2022	4,647	139	27
Cameron	2018	7,278	466	60
Comoros	2012	2,925	120	42
Dominican Republic	2013	3,255	94	29
Gambia	2019−20	7,037	361	45
Ghana	2022	3,974	105	25
Guatemala	2014−15	11,315	329	31
Haiti	2016−17	4,340	276	70
Honduras	2011−12	8,569	239	25
India	2019−21	33,019	1,260	36
Indonesia	2017	11,715	328	24
Kenya	2022	9,288	293	30
Lesotho	2023−24	1,002	31	27
Liberia	2019−20	3,578	283	78
Malawi	2015−16	14,128	663	48
Mauritania	2019−21	8,085	307	34
Myanmar	2015−16	4,571	208	44
Namibia	2013	2,514	118	45
Nepal	2022	4,633	160	33
Pakistan	2017−18	11,888	672	68
Papua New Guinea	2016−18	8,208	289	38
Peru	2012	8,769	159	18
Philippines	2022	2,423	55	20
Rwanda	2019−20	6,651	231	35
Senegal	2023	5,137	161	30
Tanzania	2022	5,211	158	31
Timor-Leste	2016	6,578	233	36
Uganda	2016	12,384	617	49
Zambia	2018	7,215	337	46
Zimbabwe	2015	4,921	251	55
**Total**	**2011-2024**	**2,66,333**	**10,978**	**40**

### Distribution and prevalence of U5M of pooled data

Table 2 summarizes the distribution and prevalence of U5M across individual- and community-level factors. A total of 266,333 children aged 0–59 months in 32 LLMICs were included in the pooled analysis. Most mothers were aged 25–34 years (50.27%), and nearly half of the children belonged to poor households (49.07%). Over half of the participants (53.98%) had media access, and 37.33% of mothers had secondary or higher education, whereas 30.29% had no formal education. Similar trends were observed for fathers’ education, with 45.89% having secondary or higher education. More than half of the children (50.40%) were born to mothers aged <19 years at first birth, and 60.10% of mothers were unemployed. The majority of births were vaginal deliveries (88.45%), and 51.33% of children were male. Two-thirds of households had ≤ 7 members (67.20%), and 64.56% of children were of birth order ≤3. Most households had improved drinking water sources (79.70%). At the community level, 68.68% of children lived in rural areas, 76.28% were in high-poverty communities, and 74.79% resided in areas with high media inaccessibility.

**Table 2:** Distribution and prevalence of U5M in 32 LLMICs, pooled DHS data, 2011–2024.

Variables	N (%)	Prevalence of U5M (95% CI) per 1,000 live births	*p-v*alue
***Individual level***			
*Total*	266,333 (100%)	40.5 (39.8-41.2)	
**Age**			<0.001
15-24	74,222 (27.87)	42.6 (41.1-44.1)	
25-34	133,878 (50.27)	37.7 (36.7-38.7)	
35-49	58,233 (21.86)	44.3 (42.6-46.0)	
**Wealth index**			<0.001
Poor	130,693 (49.07)	47.3 (46.1-48.5)	
Middle	52,855 (19.85)	41.0 (39.3-42.7)	
Rich	82,785 (31.08)	31.2 (30.0-32.4)	
**Media access**			<0.001
No	122,573 (46.02)	46.4 (45.2-47.6)	
Yes	143,760 (53.98)	35.9 (34.9-36.9)	
**Maternal education**			<0.001
No formal	80,676 (30.29)	56.0 (50.1-53.1)	
Primary	86,235 (32.38)	41.8 (40.5-43.1)	
Secondary or higher	99,422 (37.33)	31.0 (29.9-32.1)	
**Fathers’ education**			<0.001
No formal	63,239 (23.74)	49.5 (47.8-51.2)	
Primary	80,878 (30.37)	42.5 (41.1-43.9)	
Secondary or higher	122,216 (45.89)	34.7 (33.7-35.7)	
**Maternal age at birth**			<0.001
< 19	134,229 (50.40)	36.1 (35.1-37.1)	
≥ 19	132,104 (49.60)	44.8 (43.7-45.9)	
**Working status**			<0.001
Unemployed	160,074 (60.10)	38.0 (37.1-38.9)	
Employed	106,259 (39.90)	44.1 (42.9-45.3)	
**Vaginal delivery**			<0.001
Yes	235,561 (88.45)	41.4 (40.6-42.2)	
No	30,772 (11.55)	34.2 (32.2-36.2)	
**Sex of child**			<0.001
Male	136,706 (51.33)	43.9 (42.8-45.0)	
Female	129,627 (48.67)	36.9 (35.9-37.9)	
**Family member**			<0.001
≤ 7	178,983 (67.20)	41.9 (41.0-42.8)	
> 7	87,350 (32.80)	47.3 (46.0-48.6)	
**Birth order**			<0.001
≤ 3	171,932 (64.56)	35.8 (34.9-36.7)	
> 3	94,401 (35.44)	49.4 (48.0-50.8)	
**Drinking water**			<0.001
Unimproved	54,057 (20.30)	48.3 (46.5-50.1)	
Improved	212,276 (79.70)	38.7 (37.9-39.5)	
***Community level***			
**Residence**			<0.001
Rural	182,922 (68.68)	44.5 (43.6-45.4)	
Urban	83,411 (31.32)	32.1 (30.9-33.3)	
**Community poverty**			<0.001
Low	63,165 (23.72)	34.1 (32.7-35.5)	
High	203,168 (76.28)	42.7 (41.8-43.6)	
**Community maternal illiteracy**			<0.001
Low	211,896 (79.56)	39.3 (38.5-40.1)	
High	54,437 (20.44)	45.2 (43.5-46.9)	
**Community fathers’ illiteracy**			<0.001
Low	248,618 (93.35)	40.0 (39.2-40.8)	
High	17,715 (6.65)	47.1 (44.0-50.2)	
**Community media inaccessible**			<0.001
Low	67,132 (25.21)	34.0 (32.6-35.4)	
High	199,201 (74.79)	42.8 (41.9-43.7)	
**Community maternal unemployment**			<0.001
Low	9,408 (3.53)	38.4 (34.5-42.3)	
High	256,925 (96.47)	40.5 (39.7-41.3)	
**Community vaginal delivery**			<0.001
Low	3,555 (1.33)	22.3 (17.4-27.2)	
High	262,778 (98.67)	40.8 (40.0-41.6)	
**Community unimproved water sources**			0.585
Low	2,509 (0.94)	35.9 (28.6-43.2)	
High	263,824 (99.06)	40.5 (39.7-41.3)	

The overall U5M prevalence was 40.5 deaths per 1,000 live births (95% CI: 39.8–41.2). At the individual level, the highest prevalence of U5M observed among mothers aged 35–49 years (44.3 deaths per 1,000 live births). Socioeconomic disparities were evident, as children from poor households experienced the highest U5M prevalence (47.3 deaths per 1,000 live births), compared to those from rich households (31.2 deaths per 1,000 live births). Lack of maternal education was strongly associated with higher child mortality (56 deaths per 1,000 live births for mothers with no formal education vs. 31.0 deaths per 1,000 live births for mothers with secondary or higher education). Similar trends were observed for paternal education. Media inaccessibility, maternal employment, female child, large household size (>7 members), higher birth order (>3), and reliance on unimproved water sources were all linked with elevated U5M rates.

At the community level, significant geographic and socioeconomic gradients were observed. Rural children had a markedly higher U5M prevalence (44.5 deaths per 1,000 live births) compared to their urban counterparts (32.1 deaths per 1,000 live births). Communities characterized by high poverty, maternal or paternal illiteracy, and low media access reported higher U5M prevalence. Interestingly, community-level maternal unemployment showed minimal variation, whereas low community-level vaginal delivery prevalence was associated with notably reduced U5M (22.3 deaths per 1,000 live births). However, no significant difference in U5M prevalence was observed for community-level unimproved water sources (*p* = 0.585).

### Factors associated with U5M

**Fig 1:**
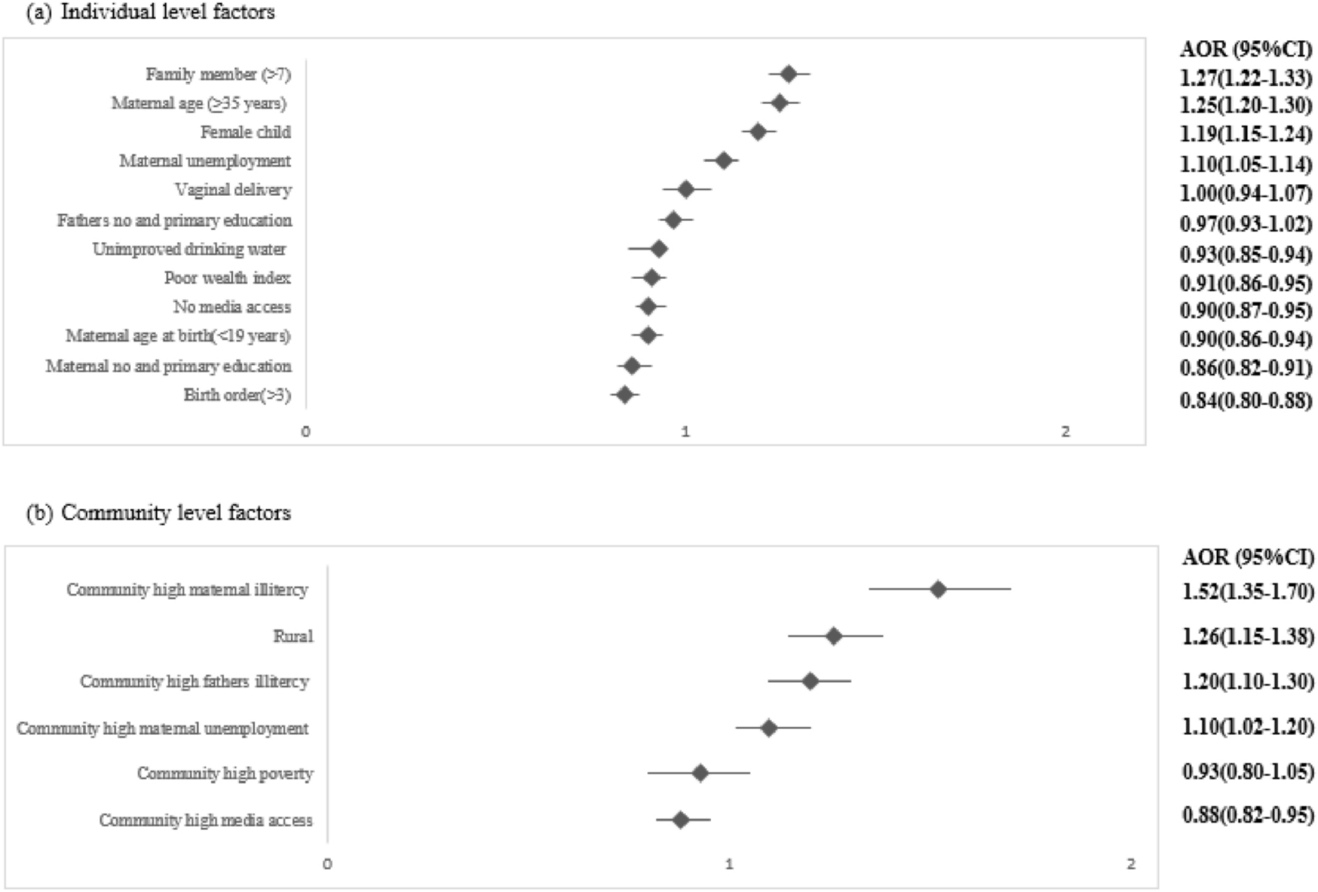
Relative importance of factors associated with under-five mortality (U5M) based on the fully adjusted model.

**Fig 1** presents the relative ranking of factors associated with U5M based on the results from binary logistic regression models at both the individual and community levels. At the individual level (Fig 1a), the leading factors associated with U5M were ranked by adjusted odds ratios (AORs). The strongest predictor was having ≥7 family members (AOR: 1.27, 95% CI: 1.22–1.33, p < 0.001), followed by maternal age ≥ 35 years (AOR: 1.25, 95% CI: 1.20–1.30, p < 0.001), female child (AOR: 1.19, 95% CI: 1.15–1.24, p < 0.001), and maternal unemployment (AOR: 1.10, 95% CI: 1.05–1.14, p < 0.001), with vaginal delivery showing a smaller but positive association. At the community level (Fig 1b), the highest-ranked factor was high maternal illiteracy (AOR: 1.52, 95% CI: 1.35–1.70, p < 0.001), followed by rural residence (AOR: 1.26, 95% CI: 1.15–1.38, p < 0.001), high paternal illiteracy (AOR: 1.20, 95% CI: 1.10–1.30, p < 0.001), and high maternal unemployment (AOR: 1.10, 95% CI: 1.02–1.20, p < 0.001). These results indicate that household size and maternal literacy are the strongest individual- and community-level determinants of U5M, respectively.

### Country-specific analysis

**Fig 2:**
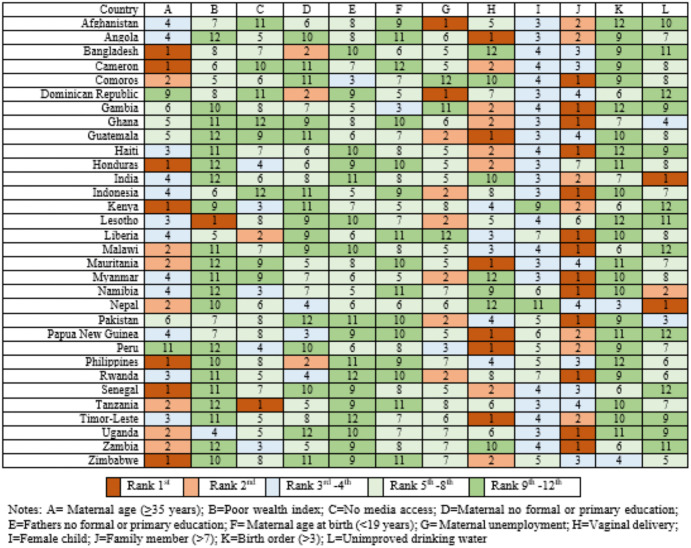
Country-specific ranking of 12 individual-level factors associated with U5M.

**Fig 3:**
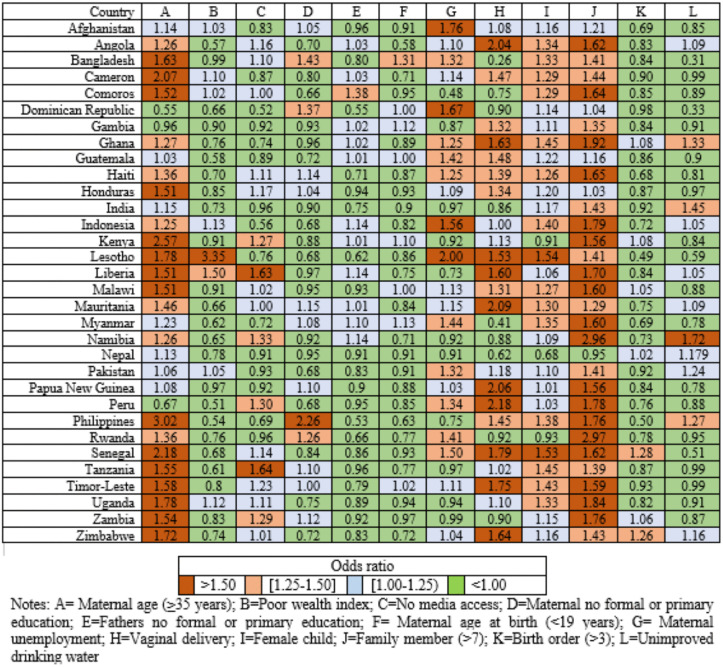
Country-specific odds ratios for 12 individual-level factors associated with U5M.

**Fig 4:**
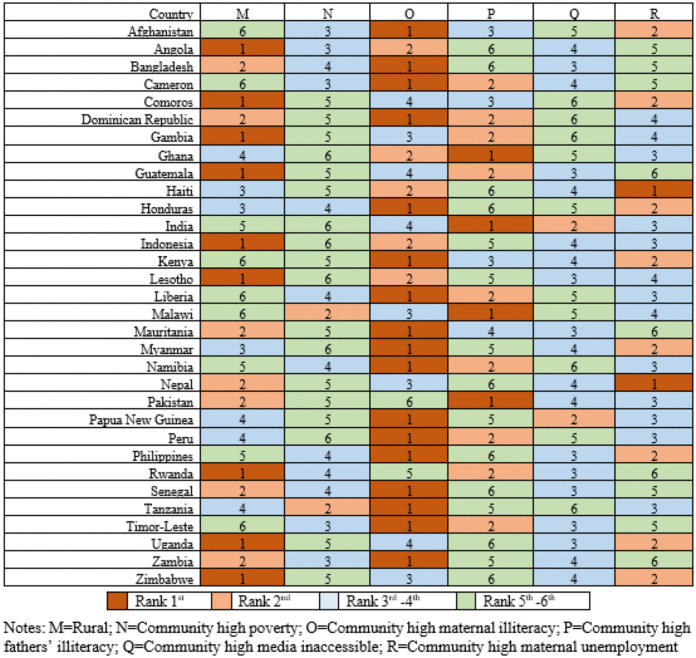
Country-specific ranking of 6 community-level factors associated with U5M.

**Fig 5:**
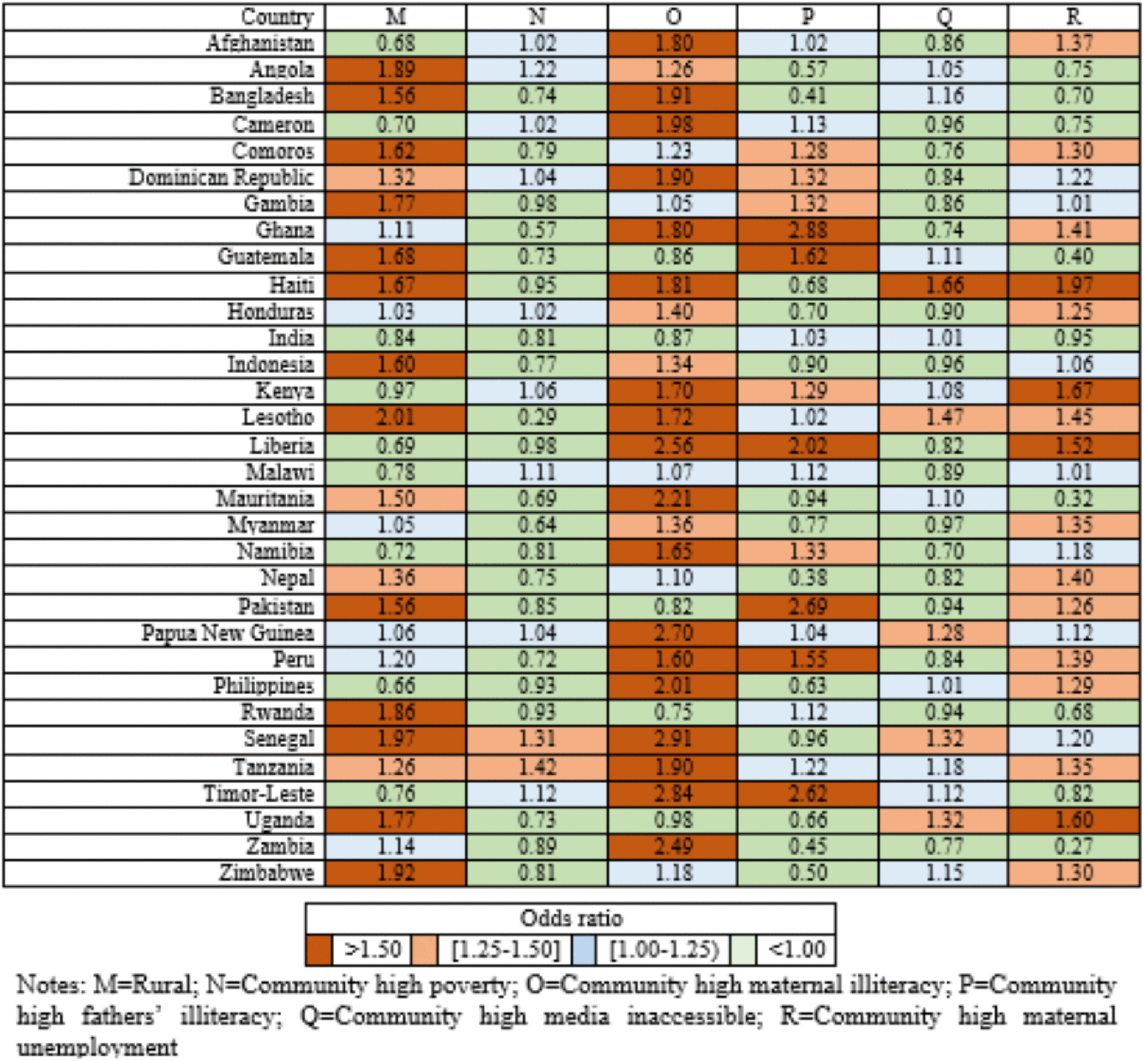
Country-specific odds ratios for 6 community-level factors associated with U5M.

Country-specific analyses at both the individual and community levels are presented in **Figs 2–5**. At the individual level, larger family size (>7 members) showed the strongest association with U5M of children across 32 LLMICs, ranking first in ORs in 13 countries and second or third in 12 others (**Fig 2**).

Maternal age of 35 years or older was also a leading factor, ranking highest in countries such as Cameron, Honduras, Kenya, Philippines, Senegal, and Zimbabwe. However, these two factors were not significant in Bangladesh. Vaginal delivery emerged as a key risk factor in several countries including Angola, Guatemala, Mauritania, Papua New Guinea, Peru, and Timor-Leste. Additionally, leading factors included poor wealth index in Lesotho, lack of media access in Tanzania, and the unimproved drinking water sources in India and Nepal. Being a female child was consistently associated with a survival disadvantage, raking near the top in several LLMICs. The adjusted odds ratios (AORs) for these factors showed considerable variation in magnitude, as illustrated in **Fig 3**. For example, the AORs for vaginal delivery ranged from 0.26 in Bangladesh to 3.35 in Lesotho for the poor wealth index.

At the community level, over half of the countries ranked maternal illiteracy as the top factor, followed by children residing in rural areas (**Fig 4**). High levels of paternal illiteracy and maternal unemployment within communities were also among the top-ranking factors in countries such as Ghana, India, Malawi, and Pakistan, Haiti, and Nepal. Due to multicollinearity, the community-level variable for vaginal deliveries was eliminated from the model. The AORs for high community-level maternal unemployment ranged from 0.27 in Zambia to 2.91 for high community-level maternal illiteracy in Senegal (**Fig 5**).

## Discussion

There is a growing global emphasis on reducing U5M and promoting childcare in resource-limited settings within LLMICs. Following the successful achievement of the Millennium Development Goals (MDGs), most LLMICs have shifted their focus toward the Sustainable Development Goals (SDGs), which include the target of reducing U5M to fewer than 5 deaths per 1,000 live births [30,45].

Pooled and country-specific analyses identified several key determinants of U5M. At the individual level, maternal age (≥35 years), large family size (>7), maternal unemployment, and vaginal delivery were all significantly associated with increased U5M. At the community level, high maternal illiteracy, rural residence, and high paternal illiteracy emerged as strong predictors of U5M.

One of the most striking findings of this study is the significant association between large family size and U5M across LLMICs. Consistent with prior research [46], increasing family size is strongly associated with a significantly higher risk of child mortality, highlighting the compounded vulnerability faced by children in larger households where limited resources, reduced parental attention, and potential competition for care may collectively contribute to poorer survival outcomes. In many low-resource settings, the financial burden of sustaining large households with limited income restricts access to adequate nutrition, healthcare, and sanitation, ultimately compromising child survival. When mothers experience inadequate nutrition during pregnancy, their children are more likely to be born with growth restrictions and face an elevated risk of illness and death [47]. Additionally, as family size grows, intra-family competition for food and essential resources intensifies, reducing both the quality of care and the amount of parental time and attention available for each child factors that collectively elevate U5M risk. Multivariate analysis also indicates that children born to mothers aged ≥ 35 years faced a significantly higher risk of U5M, which aligns with existing studies identifying advanced maternal age as a significant predictor of child mortality [23,48]. This association may reflect lower educational attainment among older mothers in many LLMICs, as well as reduced awareness of optimal childcare practices [29].

Another important finding is the significant association between vaginal delivery and U5M. Evidence from a population-based cohort study in Brazil suggests that cesarean delivery may reduce child mortality [49], while research in Bangladesh reported higher mortality rates among vaginal deliveries, especially within the first three days of life [50]. Possible explanations include birth-related complications such as scalp abrasions, cephalohematoma, cerebral hemorrhage, low birth weight, malpresentation, infections, and inadequate maternal health care all of which may contribute to higher neonatal and infant mortality [51].

Maternal employment status was also a key determinant of child survival. Children of unemployed mothers are facing a greater risk of U5M, consistent with research indicating that unemployment can heighten psychological stress, economic hardship, and reduced parenting confidence factors that negatively impact child health [40]. Interestingly, this result contrasts with some studies that have reported higher U5M among children of employed mothers, particularly those engaged in agricultural or labor-intensive work, where occupational demands may limit breastfeeding and childcare [52]. These mixed findings suggest that the impact of maternal employment on child mortality may depend on the type and conditions of employment, highlighting the need for context-specific interventions. The results of this study also revealed that female children experienced higher U5M rates in several LLMICs, despite the biological vulnerability of male children. This observed female disadvantage likely reflects socio-cultural variables, such as son preference, unequal caregiving, and restricted access to healthcare and nutrition for girls [53–55]. Recent evidence from India and multi-country analyses corroborates this pattern, linking gender-based discrimination and care-seeking bias to elevated mortality among female children [55–56].

At the community level, maternal illiteracy was consistently identified as one of the most significant factors of child mortality. Previous studies have shown that maternal education plays a critical role in improving child survival by enhancing health literacy, health-seeking behavior, and the ability to advocate for children’s needs [57–58]. Similarly, paternal illiteracy was associated with increased U5M risk, supporting earlier findings that parental education jointly shapes household health decisions and resource allocation [26,30].

Finally, the results of this study, confirmed a persistent rural–urban disparity in child mortality, consistent with earlier studies [48,59]. Children living in rural areas are more likely to die from preventable causes such as infections, malnutrition, and complications of poor sanitation, which are compounded by limited access to quality healthcare [60]. These findings underscore the need for integrated, community-level interventions targeting education, healthcare access, and socioeconomic support to reduce U5M in rural and underserved populations.

### Recommendations

Based on the findings and discussions of this study, several targeted recommendations can guide policymakers in reducing U5M. First, government and non-governmental organizations should prioritise the availability, accessibility and uptake of family planning resources. Since large family size was identified as a major risk factor for U5M, effective family planning programs can help families maintain manageable household sizes and improve resource allocation for children’s health and nutrition. Second, community-based education initiatives should be strengthened, with a particular focus on women and older adults. Education programs should emphasize maternal and child health knowledge, including nutrition, hygiene, immunization, and early care-seeking behaviors, to empower caregivers and improve household health practices. Third, given the observed association between vaginal delivery and increased child mortality, it is essential to raise awareness about safe delivery practices. Policymakers should ensure that pregnant women have timely access to skilled birth attendants and medically necessary cesarean sections, thereby minimizing complications that contribute to preventable child deaths. Finally, empowering women through workforce participation should be promoted, especially in rural and resource-limited areas. Economic independence can enhance mothers’ confidence, decision-making capacity, and access to healthcare, which collectively contribute to improved child survival outcomes.

### Strengths and limitations

This study has several notable strengths. The main strength lies in its use of a population-based, nationally representative Demographic and Health Survey (DHS) data. This study examines U5M across 32 LLMICs, providing a highly representative overview of global patterns in these settings. Furthermore, this study uniquely incorporated community-level characteristics, allowing for an assessment of their collective influence on child survival, which adds valuable insight beyond individual-level determinants.

Despite these strengths, this study has some limitations. First, the variation in sample sizes across countries may have influenced the pooled estimates. Second, several important factors were not included in the analysis due to data unavailability, such as the child body mass index (BMI), the maternal antenatal care (ANC) visits, breastfeeding history, and relevant medical histories. Finally, although this study included data from multiple countries, the overall pooled sample size may still limit the generalizability of the findings to all LLMICs.

## Conclusion

Although U5M rates in LLMICs have declined significantly in recent years, achieving the SDG target of fewer than five deaths per 1,000 live births 2030 remains unlikely without accelerated action. The findings of this study underscore the urgent need for comprehensive, multi-level interventions. Key priorities include promoting family planning to reduce household size, ensuring safe and supportive working environments for women, and implementing policies aimed at eliminating gender-based discrimination. Equally critical is the expansion of women’s education, which has consistently been shown to improve child survival outcomes. Strengthening health systems, addressing structural inequities, and fostering community engagement will be crucial for accelerating progress toward global child survival goals.
